# Necrotizing fasciitis of the thigh due to penetrated descending colon cancer: a case report

**DOI:** 10.1186/s40792-018-0544-y

**Published:** 2018-11-26

**Authors:** Kentaro Sato, Hitoshi Yamamura, Yoshiyuki Sakamoto, Hajime Morohashi, Takuya Miura, Toru Yoshikawa, Akiko Suto, Satoru Tsuruta, Kenichi Hakamada

**Affiliations:** 1 Department of Gastroenterological Surgery, Hirosaki University Graduate School of Medicine, 5 Zaifu-cho, Hirosaki, Aomori, 036-8562 Japan; 2 Department of Disaster and Critical Care Medicine, Hirosaki University Graduate School of Medicine, 5 Zaifu-cho, Hirosaki, Aomori, 036-8562 Japan

**Keywords:** Necrotizing fasciitis, Colon cancer, Descending colon cancer, One-stage resection, Radical resection

## Abstract

**Background:**

Necrotizing fasciitis (NF) caused by colorectal cancer is rare, and very few cases associated with colon cancer have been reported. We describe the case of a patient with NF in the left thigh due to penetration of descending colon cancer who was treated with one-stage surgical resection without creating a stoma.

**Case presentation:**

An 80-year-old woman was brought to our hospital complaining of fever and difficulty with body movement. A physical examination showed subcutaneous emphysema from the left lower abdomen to the left femoral region. CT showed abscess formation with emphysema around the descending colon, and the wall of the descending colon was thickened, which led to suspicion of colon cancer. The patient was subsequently diagnosed with NF due to penetration of descending colon cancer. Left hemicolectomy and open drainage of the left femoral region was performed. The histopathological findings were well-differentiated adenocarcinoma, with the tumor grown through the serosa (T4a) and with no metastasis to lymph nodes (N0). After surgery, the patient received intensive care for septic shock and lavage of the open drainage site, and sepsis was controlled progressively. After closure of the drainage site, the patient was transferred to a different hospital at 26 days after surgery, and she has had 6-month relapse-free survival.

**Conclusions:**

In NF caused by colon cancer, early one-stage resection may improve the oncological outcome. Physical status should be assessed carefully, and one-stage resection should be considered if the patient has the capacity to undergo this procedure.

## Background

Necrotizing fasciitis (NF) is a progressive infection in the fascial planes with necrosis of the subcutaneous tissue [1]. The most common causes of NF are trauma, urinary tract disease, and perineal abscess [2]. NF of the perineum and genitalia is referred to as Fournier’s gangrene (FG) [3], and there are some reports of FG caused by rectal cancer [3]. However, very few cases of NF associated with colon cancer have been reported. Here, we describe a case of NF of the left thigh due to penetration of descending colon cancer that was treated with one-stage resection of the primary tumor and open drainage without creation of a diverting stoma.

## Case presentation

An 80-year-old woman was brought to our hospital complaining of fever and difficulty with body movement. Her medical and surgical histories were unremarkable. She had a body temperature of 37.7 °C, blood pressure 147/113 mmHg, heart rate 124 beats/min, and respiration rate 17/min. On physical examination, subcutaneous emphysema was found from the left lower abdomen to the left femoral region. Muscular defense was not found. Blood tests revealed an elevated white blood cell (WBC) count and C-reactive protein (CRP) level, and decreased hemoglobin (Hb) and platelet count. The examination also revealed acute kidney injury. Blood glucose was 137 mg/dL, and HbA1c was 6.4% (Table 1). On the first day, the sequential organ failure assessment (SOFA) score was 1, and the Quick SOFA score was 0.

**Table 1:** Blood biochemistry at admission

Blood analysis				Biochemical examination			
WBC	40,500	/μL	↑	TP	5.6	g/dL	↓
RBC	318 × 10^4^	/μL	↓	Alb	1.6	g/dL	↓
Hb	6.4	g/dL	↓	T-bil	0.9	mg/dL	↑
Hct	20.5	%	↓	AST	91	U/L	↑
Plt	5.9 × 10^4^	/μL	↓	ALT	45	U/L	↑
				LDH	240	U/L	↑
Congealing fibrinogenolysis system				CPK	716	U/L	↑
PT	17.3	s		BUN	70	mg/dL	↑
APTT	43.2	s		Cre	1.42	mg/dL	↑
FDP	6.1	μg/mL	↑	Na	133	mmol/L	↓
D-dimer	1.5	μg/mL	↑	K	4.3	mmol/L	
				Cl	95	mmol/L	↓
				CRP	55.9	mg/dL	↑
				BS	137	mg/dL	↑
				HbA1c	6.4	%	↑

**Fig. 1:**
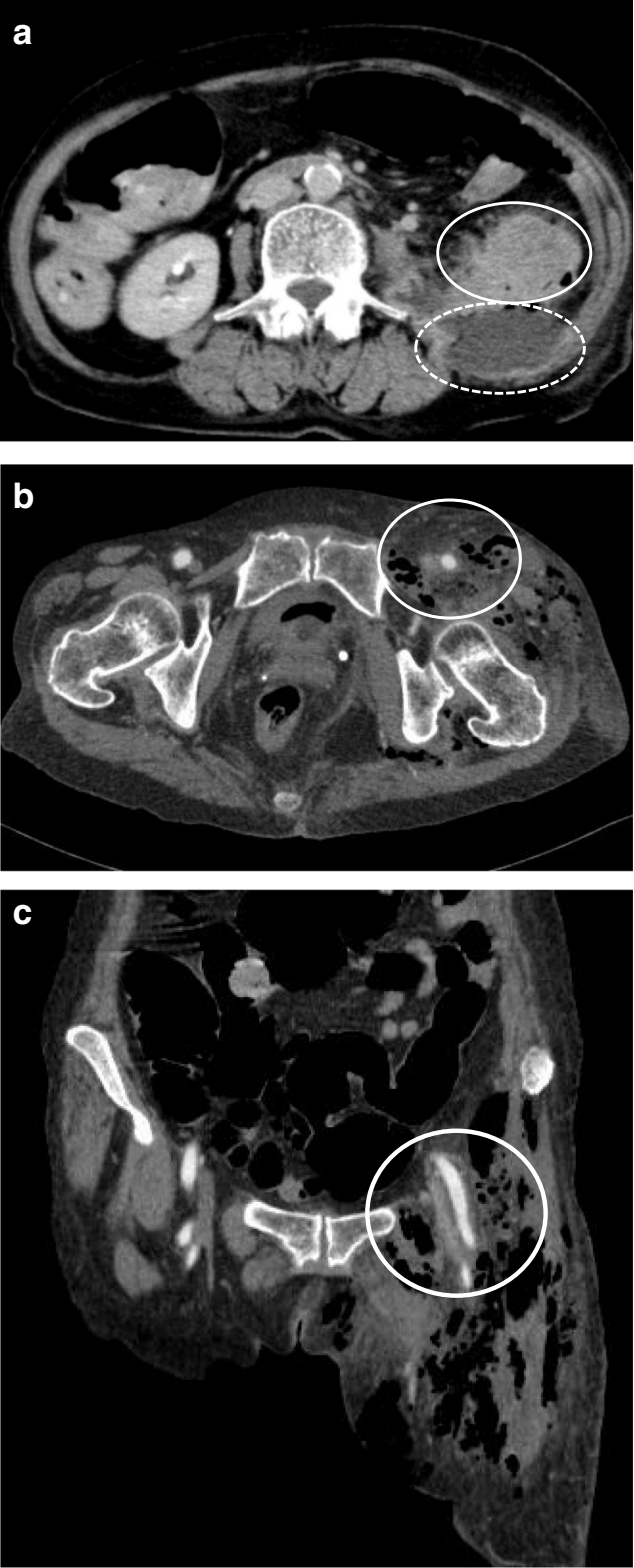
**a** Computed tomography showed a thickened wall of the descending colon (circle) and abscess formation around this area (dotted circle). **b**, **c** Emphysema was present from the retroperitoneal abscess around the descending colon to the left thigh through the left femoral region (circles)

Enhanced computed tomography (CT) showed a thickened wall of the descending colon and retroperitoneal abscess formation in this area. From these findings, penetrating descending colon cancer was suspected (Fig. 1a). Emphysema was present from the retroperitoneal abscess around the descending colon to the left thigh through the left femoral ring (Fig. 1b, c). Lymph node metastasis and distant metastasis were not detected.

**Fig. 2:**
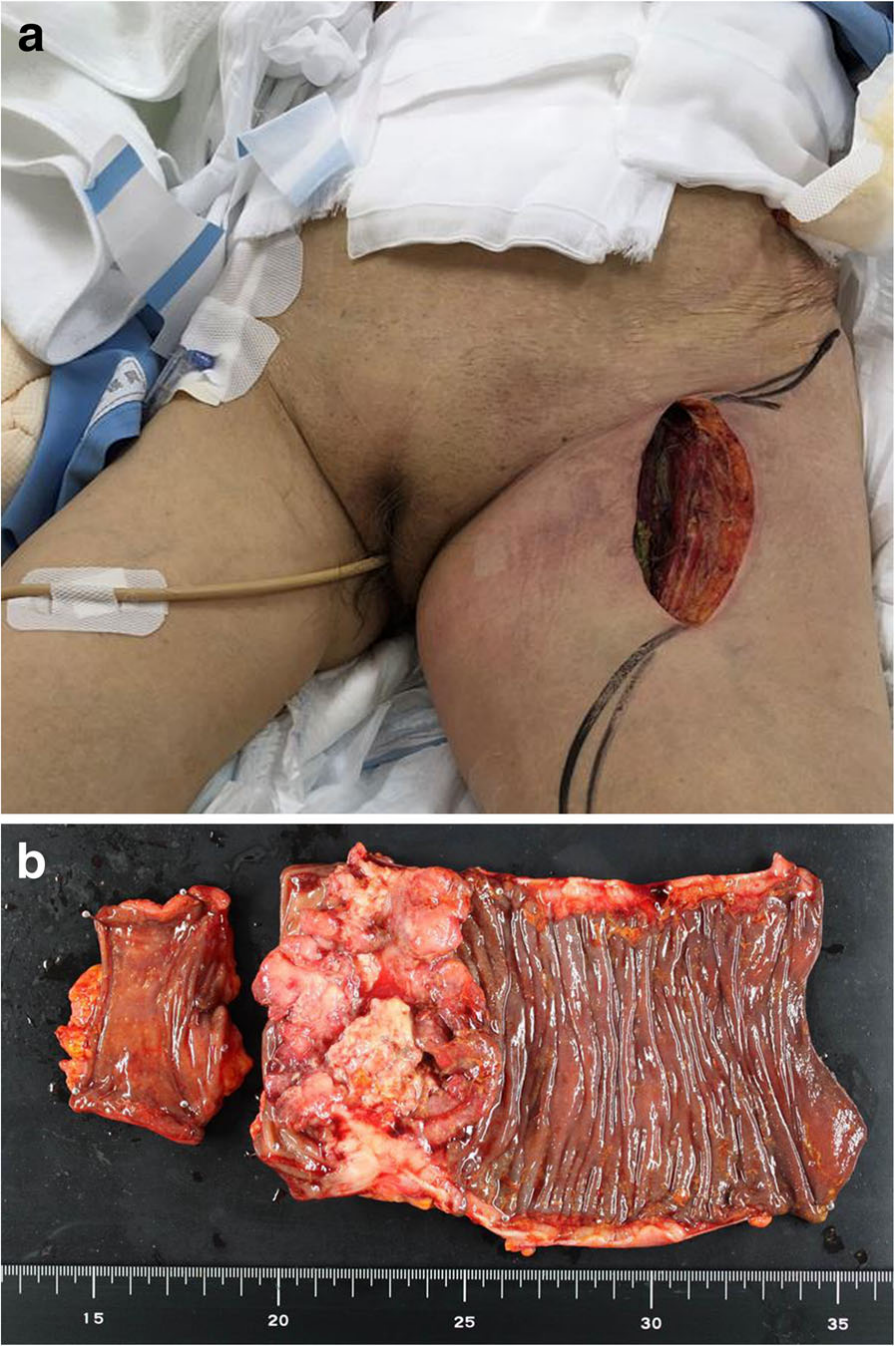
**a** Open wound for drainage of the peritoneum and left thigh. **b** Surgical resected specimen of left hemicolectomy revels that tumor invades into the serosa (T4a) with no regional lymph node metastasis (N0)

The patient was suspected to have NF due to penetration of descending colon cancer. Left hemicolectomy and open drainage of the left femoral region were performed (Fig. 2a, b). A microbiological culture of the abscess revealed the presence of group C β-*Streptococcus*, *Escherichia coli*, *Prevotella* species, and *Corynebacterium* species. Histopathological findings showed a tumor with a histological type of well-differentiated adenocarcinoma. The tumor had grown through the serosa (T4a), but there was no metastasis to lymph nodes (N0).

**Fig. 3:**
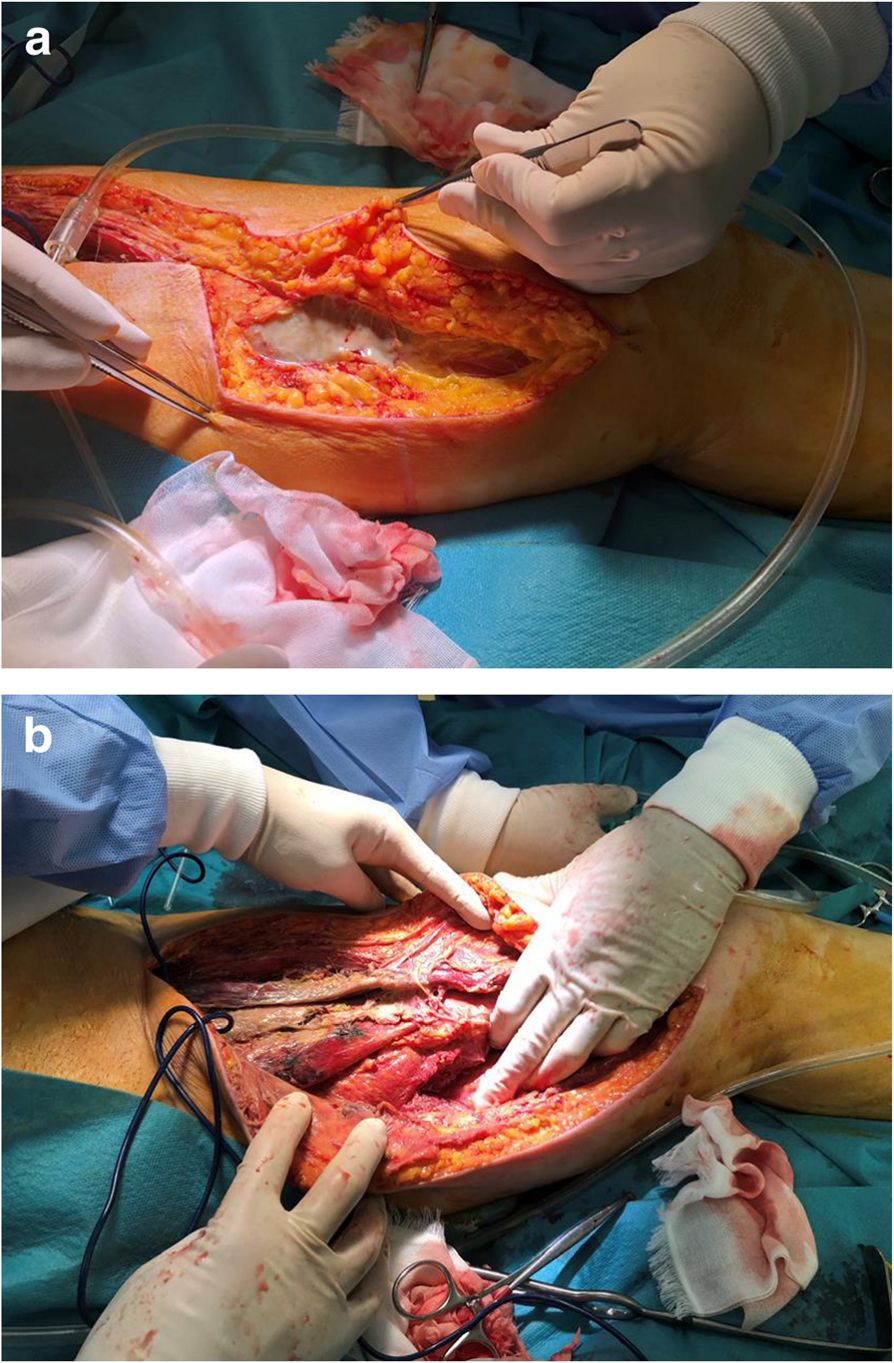
**a**, **b** Additional extensive open wound for drainage from the initial drainage site of the left thigh (Fig. 2a) to the level of the left knee was performed on day 1 after surgery

After surgery, the patient received intensive care for sepsis and underwent lavage of the open drainage site. Drainage was insufficient, and so additional open drainage was performed on postoperative day (POD) 1 (Fig. 3a, b). After additional drainage, control of focal infection was successful and sepsis was gradually controlled. Follow-up CT on POD 11 showed no residual abscess, and so negative pressure wound therapy was tried. Closure of the drainage site was performed on POD 22, and the patient was transferred to a different hospital on POD 26. She has now achieved 6-month relapse-free survival.

## Discussion and conclusions

NF can easily cause severe sepsis and disseminated intravascular coagulation (DIC), and thus, the mortality rate is high (25–75%) even with appropriate treatment [1]. In a review of 1726 cases of FG, Eke found a mortality rate of 16% [4]. NF due to colorectal cancer is rare and most cases are FG caused by rectal cancer. In Japan, 17 cases of FG caused by rectal cancer have been reported [5] and the prognosis of these cases is unsatisfactory, with only 3 having survival of more than 1 year. There are several reports of NF and FG due to colon cancer involving the abdominal wall [6–10], whereas NF of the thigh due to a retroperitoneal abscess caused by colorectal cancer, as in our case, is extremely rare. To our knowledge, only 5 such cases have been described, including the present case [11–14] (Table 2), and only 3 of these are due to colon cancer. In these cases, a retroperitoneal abscess formed through the femoral ring and reached the thigh, causing NF to occur in the thigh.

**Table 2:** Cases of necrotizing fasciitis associated with colorectal cancer

Case (ref.)	Author	Year	Age	Sex	Tumor location	Therapy	Stoma	Prognosis
1 (11)	Lam	1996	67	Female	Sigmoid colon	One-stage resection and open drainage	End colostomy	Death (3 days)
2 (12)	Liu	2006	56	Male	Rectum	Open drainage	Not created	Death (6 days)
3 (13)	Highton	2008	79	Male	Rectum	Two-stage resection after drainage and colostomy	End colostomy	Alive (survival time unknown)
4 (14)	Takakura	2009	67	Male	Sigmoid colon	Two-stage resection after drainage	Not created	Death (8 months)
5	Our case	2018	80	Female	Descending colon	One-stage resection and open drainage	Not created	Alive (6 months RFS)

The main treatment for NF due to colorectal cancer is surgical therapy and intensive care for sepsis. In most cases, the initial operation is open drainage, and in a case of rectal cancer, a colostomy is often created [5]. After NF is controlled, the primary tumor is resected. Ishibashi et al. [15] reported that cases of FG with ≤ 12 h from diagnosis to surgery had a higher survival rate, and so the initial operation should be performed immediately. In most cases of FG with rectal cancer, a diverting or permanent colostomy is created to avoid contamination of the perineum by stool and to allow early oral ingestion. In our case, the tumor was located in the descending colon and we performed left hemicolectomy without creation of a diverting stoma.

In NF due to colon cancer, the need for a stoma is controversial. If the mortality risk is high, a creating stoma should be considered prior to lifesaving surgery. In NF, the main cause of death is severe sepsis, so the SOFA score [16] may also be useful to predict the mortality risk. In our case, the SOFA score was low and the patient did not have severe septic shock. Therefore, we thought that performance of one-stage resection and intestinal anastomosis was possible. Furthermore, NF was localized in the left thigh and did not reach the perineum, so we decided that a stoma was not necessary. If creating a stoma is required in a case of colon cancer, a diverting stoma should be chosen when possible to preserve future anal function.

In most cases of NF and FG, progression of colorectal cancer is advanced [17]. To achieve a good oncological outcome, early oncological therapy such as surgical resection and chemotherapy is necessary. In our case, we achieved relapse-free survival for 6 months by performing one-stage resection. We will continue to check for recurrence, but the initial results suggest that early one-stage resection may improve the oncological outcome. We also attempted negative pressure wound therapy from POD 11. Kuroda et al. [18] reported the utility of this therapy for NF and FG for controlling of the focal infection, control of exudates, promotion of hyperplasia of granulation tissue, and maintaining a wet environment. All these effects promote wound healing, which suggests that negative pressure wound therapy is a good indication for NF and FG because of the large open wound and long duration of therapy.

In conclusion, we have described a rare case of NF in the left thigh due to penetration of descending colon cancer. Early open drainage and one-stage surgical resection of the tumor without stoma creation was effective in this case. In NF caused by colon cancer, creating a stoma should be considered carefully based on a detailed assessment of the patient’s physical status, and if possible, use of a stoma should be avoided.

## Abbreviations

FG: Fournier’s gangrene
NF: Necrotizing fasciitis
SOFA: Sequential organ failure assessment
